# Delayed Hypersensitivity Reaction to Infliximab Due to Mammalian Meat Allergy

**DOI:** 10.1097/PG9.0000000000000322

**Published:** 2023-06-09

**Authors:** Esthermari González Polanco, Stephen Borowitz

**Affiliations:** From the * Division of Pediatric Gastroenterology, Hepatology, and Nutrition, Department of Pediatrics, University of Virginia, Charlottesville, USA.

**Keywords:** mammalian meat allergy, inflammatory bowel disease, Infliximab, alpha-gal

## Abstract

Mammalian meat allergy is a delayed immunoglobulin E (IgE) mediated hypersensitivity reaction to galactose-alpha-1,3-galactose (alpha-gal). Alpha-gal is an oligosaccharide present on glycoproteins and glycolipids of nonprimate mammals as well as biologic agents prepared using mammalian cells including infliximab. We describe a pediatric patient with Crohn’s disease who developed urticaria and pruritus roughly 6 hours after her very first infliximab infusion that progressed to chronic urticaria following subsequent infliximab infusions. She was diagnosed with mammalian meat allergy based on an elevated serum IgE level directed against alpha-gal. Her symptoms resolved once infliximab infusions were discontinued and did not recur after commencing therapy with adalimumab.

## INTRODUCTION

Mammalian meat allergy (MMA) is an immunoglobulin E (IgE)-mediated hypersensitivity reaction to the oligosaccharide galactose-alpha-1,3- galactose (alpha-gal) found on numerous glycoproteins and glycolipids of nonprimate mammals. Alpha-gal is present in numerous food products as well as biologic agents and vaccines prepared using mammalian cells (1). In the United States, sensitization to alpha-gal usually occurs following a bite from the lone star tick (*Amblyomma americanum).* The lone star tick is widely distributed throughout the eastern and southeastern United States and its range appears to be increasing (2). People suffering from MMA typically present with recurrent episodes of urticaria, angioedema, and/or anaphylaxis, however, affected people can also experience severe abdominal cramping with vomiting and diarrhea. Unlike with most IgE-mediated food allergies where urticaria, laryngeal edema, bronchospasm, and/or vomiting, and diarrhea develop within minutes of ingestion, the symptoms of MMA typically develop 3–8 hours after consuming alpha-gal-containing foods. Reactions most commonly occur after ingesting beef, pork, or lamb but can also occur after ingesting dairy products or gelatin (3). The diagnosis of MMA is based on characteristic clinical symptoms and an elevated serum IgE level directed against alpha-gal (>0.1 IU/ml) (3).

Alpha-gal is also found on a number of monoclonal antibodies. This was first identified with the antineoplastic agent cetuximab when a number of patients in the Southeastern United States developed anaphylactic reactions to cetuximab due to MMA (4). Cetuximab is derived in a mouse-derived SP2/0 cell line resulting in alpha-gal-containing glycosylation of the antigen-binding (Fab) region of the molecule (5). A number of other monoclonal antibodies including infliximab are derived in the same cell line and thus are glycosylated with alpha-gal (5). The risk of anaphylaxis to infliximab in patients with known MMA has been thought to be relatively low not only because infliximab has significantly lower alpha-gal content than cetuximab, but also because much of the alpha-gal on infliximab is on the crystallizable fragment (Fc) region of the antibody which interacts with cell surface receptors rather than the antibody binding region of the molecule (5). Nevertheless, some patients with a known history of MMA demonstrate increased production of IgE directed against alpha-gal when they are exposed to infliximab (6).

## CASE PRESENTATION

AP is a 17-year-old female who was diagnosed with ileal and perianal Crohn’s disease after presenting with 4 months of abdominal pain and bloody and mucousy diarrhea. After pretreatment with diphenhydramine and acetaminophen per our institutional protocol, she received her first infusion of 10 mg/kg of infliximab and she tolerated the infusion without any adverse effects. Approximately 6 hours after completing her first infliximab infusion, she developed an extremely pruritic urticarial eruption behind her ears, over her neck, and on her back. She had neither difficulty breathing nor any abdominal pain, nausea, vomiting, or diarrhea. She was treated with oral diphenhydramine and the urticaria and pruritus resolved over several hours. With each subsequent infusion of infliximab, she was pretreated with diphenhydramine and acetaminophen, and she tolerated the infusions without any adverse effects however between 4 and 6 hours after infusions were completed, she developed an urticarial eruption in a variable distribution. The episodes of urticaria and pruritus were always brief and resolved following the administration of oral diphenhydramine and she had no other associated symptoms. Her Crohn’s disease responded well to the infliximab and at her fourth infusion, she had experienced complete resolution of her abdominal symptoms and a fecal calprotectin was <5 mcg/g of stool. Following her fifth infusion, the urticaria and pruritus were more persistent and did not resolve with administration of diphenhydramine and so she was treated with a short course of oral corticosteroids. Two weeks after her fifth infliximab infusion, an infliximab level was 25 mcg/ml and no antidrug antibodies were identified. Complete blood count demonstrated a white blood cell count of 6900 with 1.3% eosinophils. Total serum IgE was 28.2 IU/ml (nl 10.0–180.0) however, IgE directed against alpha-gal was 1.60 kU/L (nl <0.35 kU/L) and comprised ~6% of total IgE which is highly significant (7). The patient was an avid cross-country runner and did most of her running in rural Virginia and she had a history of numerous tick bites. She was a lacto-ovo-vegetarian and had not been eating any mammalian meat; however, she was instructed to eliminate all dairy products as well as products containing gelatin from her diet. Infliximab infusions were discontinued and she was switched to adalimumab. Her chronic urticaria resolved over several weeks and did not recur even after she resumed eating dairy products and her Crohn’s disease remained in remission on adalimumab monotherapy. Four months after switching from infliximab to adalimumab her IgE levels directed at alpha-gal had declined from 1.60 kU/L to 0.23 kU/L.

## DISCUSSION

While infusion reactions to infliximab are not unusual, these reactions typically occur during the infusion or within 2 hours of completing the infusion, and delayed reactions are quite rare. When delayed reactions occur, they are typically serum sickness reactions that develop more than 24 hours after the infusion (8). The fact that the patient developed urticaria and pruritus approximately 6 hours after her very first infliximab infusion is strongly suggestive of preexisting IgE directed against alpha-gal that interacted with the infliximab. Furthermore, she had no history of urticaria before getting infliximab infusions, and her symptoms completely resolved and have not recurred after discontinuing infliximab infusions and switching to adalimumab. Adalimumab is a recombinant human IgG1 monoclonal antibody derived in a different cell line than infliximab and thus adalimumab is not glycosylated with alpha-gal.

MMA causing allergic reactions to infliximab appears to be uncommon. However, this disorder should be considered in any patient who experiences hypersensitivity reactions to infliximab, particularly when the reaction occurs after the first infusion and/or the reaction does not occur during the infusion, and rather is delayed by a number of hours. Furthermore, among patients with a known history of MMA, biologic agents that are glycosylated with alpha-gal should be avoided whenever possible.

## ACKNOWLEDGEMENTS

The authors wish to acknowledge A.P. and her family for graciously allowing us to publish this case report.

The patient and family assented and consented, respectively, to this case report.

## References

[1] Steinke JW Platts-Mills TA Commins SP. The alpha-gal story: lessons learned from connecting the dots. J Allergy Clin Immunol. 2015;135:589–596. 25747720 10.1016/j.jaci.2014.12.1947 PMC4600073

[2] Patel C Iweala OI. “Doc, will I ever eat steak again?” – diagnosis and management of alpha-gal syndrome. Curr Opin Pediatr. 2020;32:816–824. 33009122 10.1097/MOP.0000000000000955 PMC7737235

[3] Commins P. Diagnosis and management of Alpha-GAL syndrome: lessons from 2,500 patients. Expert Rev Clin Immunol. 2020;16:667–677. 32571129 10.1080/1744666X.2020.1782745 PMC8344025

[4] Chung CH Mirakhur B Chan E. Cetuximab induced anaphylaxis and IgE specific for galactose-alpha-1,3-galactose. N Engl J Med. 2008;358:1109–1117. 18337601 10.1056/NEJMoa074943 PMC2361129

[5] Van Bueren J Rispens T Verploegen S. Anti-Galactose-α-1,3-Galactose IGE from allergic patients does not bind α-Galactosylated Glycans on intact therapeutic antibody FC DOmains. Nat Biotechnol. 2011;29:574–576. 21747378 10.1038/nbt.1912

[6] Chitnavis M Stein DJ Commins S. First-Dose anaphylaxis to infliximab: a case of mammalian meat allergy. J Allergy Clin Immunol Pract. 2017;5:1425–1426. 28634101 10.1016/j.jaip.2017.04.044

[7] Platts-Mills TAE Li RC Keshavarz B. Diagnosis and management of patients with the alpha-gal syndrome. J Allergy Clin Immunol Pract. 2020;8:15.e1–123e1. 31568928 10.1016/j.jaip.2019.09.017 PMC6980324

[8] Lichtenstein L Ron Y Kivity S. Infliximab–related infusion reactions. J Crohns Colitis. 2015;9:806–815. 26092578 10.1093/ecco-jcc/jjv096 PMC4558633

